# Hip, knee and revision hip replacement – are they as clinically and cost effective as we think?

**DOI:** 10.1080/17453674.2020.1763579

**Published:** 2020-05-12

**Authors:** Ashley Blom

**Affiliations:** Head of Bristol Medical School, University of Bristol, UK

Modern medicine is expensive and thus societal choices around resource allocation are important to ensure maximal benefit. Total hip and knee replacement are two of the commonest elective surgical treatments performed in the developed world and have been shown to be effective in relieving pain and improving function in those with advanced osteoarthritis. However, treatments need to be both clinically effective and cost effective in order to justify their widespread use, particularly in healthcare systems that are subsidised by general taxation, as is the practice in most European countries.

Comparing different treatments for different conditions is both difficult and contentious. Quality adjusted life years (QALYs) is one method of doing this and is predicated on the idea that the treatment cost of delivering a quantifiable unit of improvement in health can be measured. This will allow us to both contrast the value of treatments and ascertain levels of “willingness to pay” for health gain.

Rasanen and colleagues from Finland measured the quality of life gained following both primary and revision hip replacement and primary knee replacement by cost incurred to achieve that gain. Primary hip and knee replacement improved the mean quality of life scores of patients, but improvements after revision hip replacement were neither clinically nor statistically significant. Furthermore, the cost per unit of improvement was twice as high for TKR as THR and nearly eight times as high for revision compared to primary total hip replacement.

This paper is a landmark as it elegantly demonstrates the effectiveness and cost effectiveness of both hip and knee replacements, whilst showing what many surgeons suspected that knee replacement results in slightly lower gains, compared to hip replacements, at higher costs. Probably the most interesting finding is that revision hip replacement results in little or no improvement in overall quality of life despite being very expensive. This finding should cause surgeons to reflect on their practice. Certainly, many revisions are performed to prevent symptoms worsening (such as after fracture or infection) and this analysis compares before and after rather than the sequelae of treatment versus no treatment. However, many revisions, particularly after knee replacement, are performed to treat stable conditions such as persistent pain. Even though the work presented here was published in 2007, we as a community have not yet established the utility, efficacy and wisdom of performing revision arthroplasty for stable conditions affecting pain and function. The findings of Rasanen and colleagues are as pertinent today as they were in 2007.

Ashley Blom Head of Bristol Medical School, University of Bristol, UK E-mail: Ashley.Blom@bristol.ac.uk

